# Acarbose Use and Liver Injury in Diabetic Patients With Severe Renal Insufficiency and Hepatic Diseases: A Propensity Score-Matched Cohort Study

**DOI:** 10.3389/fphar.2018.00860

**Published:** 2018-08-07

**Authors:** Chia-Ter Chao, Jui Wang, Jenq-Wen Huang, Kuo-Liong Chien

**Affiliations:** ^1^Department of Medicine, National Taiwan University Hospital BeiHu Branch, Taipei, Taiwan; ^2^Nephrology Division, Department of Internal Medicine, National Taiwan University Hospital, Taipei, Taiwan; ^3^Geriatric and Community Medicine Research Center, National Taiwan University Hospital BeiHu Branch, Taipei, Taiwan; ^4^College of Public Health, Institute of Epidemiology and Preventive Medicine, National Taiwan University, Taipei, Taiwan

**Keywords:** acarbose, chronic kidney disease, diabetes mellitus, drug-induced liver injury, end-stage renal disease, hepatotoxicity

## Abstract

**Background:** Acarbose has been deemed contraindicated in diabetic patients with chronic kidney disease (CKD) or end-stage renal disease (ESRD), but such use is not uncommon. We tested whether this concept hold true in this population with different background hepatic diseases.

**Methods:** All incident diabetic patients (*n* = 2,036,531) with stage 5 CKD/ESRD were enrolled from Taiwan between 2017 and 2013 and divided into those without chronic liver disease (CLD), with CLD but without cirrhosis, and those with cirrhosis. Among each group, acarbose users, defined as cumulative use >30 days within the preceding year, were propensity-score matched 1:2 to non-users. Our main outcome was the development of liver injury events during follow-up.

**Results:** Acarbose users did not exhibit an increased incidence of liver injury during follow-up compared to non-users (hazard ratio and 95% confidence interval, 1.04 [0.88–1.25], 0.97 [0.61–1.56], and 0.71 [0.33–1.54] among those without CLD, with CLD but without cirrhosis, and those with cirrhosis, respectively), after adjusting for demographic profiles, comorbidities, potentially hepatotoxic medication use, and diabetic severity.

**Conclusions:** The incidence of liver injury did not increase significantly among diabetic acarbose users with severe renal insufficiency than non-users, regardless of the presence or absence of chronic liver disease. Our findings support the renaissance of acarbose as a useful adjunct in diabetic patients with stage 5 and 5D chronic kidney disease.

## Introduction

The prevalence of diabetes mellitus (DM) has continuously increased worldwide, and the number of affected patients is expected to exceed 600 million by 2,040 (Forouzanfar et al., 2016). This epidemic of DM can be attributed to population aging, dietary preferences, lifestyle alterations, and even genetic/epigenetic predisposition (Gaulton, 2017). A major complication of DM is renal dysfunction, consisting of albuminuria, chronic kidney disease (CKD), or end-stage renal disease (ESRD), and renal failure accounts for 10–20% of deaths among patients with DM (Afkarian et al., 2016). For diabetic patients developing CKD, dosage adjustment is frequently needed for their anti-diabetic regimens, due to the fact that drugs and their metabolites frequently accumulate in patients with CKD, predisposing them to adverse events. Conventional oral anti-diabetic agents, such as metformin, are contraindicated in patients with eGFR levels below 30–45 ml/min/1.73 m^2^, whilst sulfonylurea carries the risk of prolonged hypoglycemia among these patients. Options of oral anti-diabetic agents (OADs) for diabetic patients with advanced CKD are thus limited; according to the updated guideline from the American Diabetic Association, only meglitinides, thiazolidinediones, and dipeptidyl peptidase-4 inhibitors (DPP4i) are suitable for oral glucose reduction in patients with an eGFR of <30 ml/min/1.73 m^2^ (American Diabetes Association, 2018). However, each of the above OADs has its own drawbacks, including meglitinide-related hypoglycemia and myocardial infarction (Lin et al., 2016; Wu et al., 2017b), thiazolidinediones-related heart failure exacerbation and edema, and the high cost associated with DPP4i (Roussel et al., 2015). Although insulin may be the treatment of choice for these patients, studies have suggested that OAD users in combination with insulin had a significantly lower mortality than those using insulin alone (Hsiao et al., 2017). Consequently, it is imperative that our armamentarium of OADs against DM be expanded to allow safer approaches for glycemic control in diabetic CKD/ESRD patients.

Acarbose, a competitive inhibitor of intestinal α-glucosidase, reduces post-prandial carbohydrate digestion and absorption, effectively lowering glycated hemoglobin. Apart from common side effects like gastrointestinal disturbances, early trials involving acarbose reported increases in transaminases or rarely jaundice in 3.8% of users compared to 1.1% in the placebo group (Balfour and McTavish, 1993). This is attributed predominantly to the accumulation of acarbose and its metabolites, but little evidence exists to support such idea. Following the wide spread use of acarbose in DM patients more than two decades ago, case reports of acarbose-related liver injury started to emerge. The first case was a 65-year-old lady with type 2 DM receiving acarbose 300 mg/day for 2 months (Andrade et al., 1996). The predominant manifestations were malaise and anorexia, accompanied by laboratory changes of hepatocellular damage pattern, with spontaneous recovery after discontinuing acarbose. Subsequent case series suggested that reports of acarbose-related liver toxicity mostly came from Japan and Spain, had an average latency of 2–6 months after therapy initiation, frequently recurred after re-challenge, and the associated laboratory abnormalities were hepatocellular injury predominantly (Hsiao et al., 2006). In contrast to traditional hepatotoxic medications such as acetaminophen and ethanol, whose liver toxicity is mediated through hepatic microsomal enzyme activation, acarbose may not cause hepatotoxicity through its metabolites. Putative mechanisms include the induction of CYP2E1 enzyme and increasing reactive oxygen species production and the influence of sex hormone, but the exact mechanism still remains unclear due to the low and unpredictable incidence of such injury (Wang et al., 1999; Hsiao et al., 2006).

Given the lack of experiences of using acarbose in patients with advanced CKD/ESRD, and the pharmacokinetic concerns in these patients (Charpentier et al., 2000), acarbose has been contraindicated in this population (International Diabetes Federation Guideline Development Group, 2014; American Diabetes Association, 2018). However, whether these recommendations hold true decades after acarbose enters the market is unclear. We hypothesized that acarbose use might not be associated with an increased risk of liver toxicity among diabetic patients with severe renal insufficiency. Since acarbose has not been reported as being significantly removed by dialysis in the existing literature, we think that the dosage recommendation for acarbose will not differ between those with non-dialysis stage 5 CKD and dialysis-dependent ESRD. Furthermore, there is currently no evidence suggesting that dialysis influences the risk of developing hepatotoxicity. Thus, we chose these patients who traditionally have the fewest options of OADs, for analyzing whether acarbose use was associated with an increased risk of developing liver injury, using a retrospective population-based cohort approach. To avoid the possibility of effect dilution by the presence or absence of liver disease in these patients, we planned to analyse whether this association appeared in participants without or with different severities of liver diseases.

## Methods

### Ethical approval

The current study has been approved by the institutional review board of National Taiwan University Hospital (No. 201503028W), and its protocol adheres to the declaration of Helsinki. Informed consent was waived due to data anonymity.

### Participant enrollment and data sources

**Figure 1:**
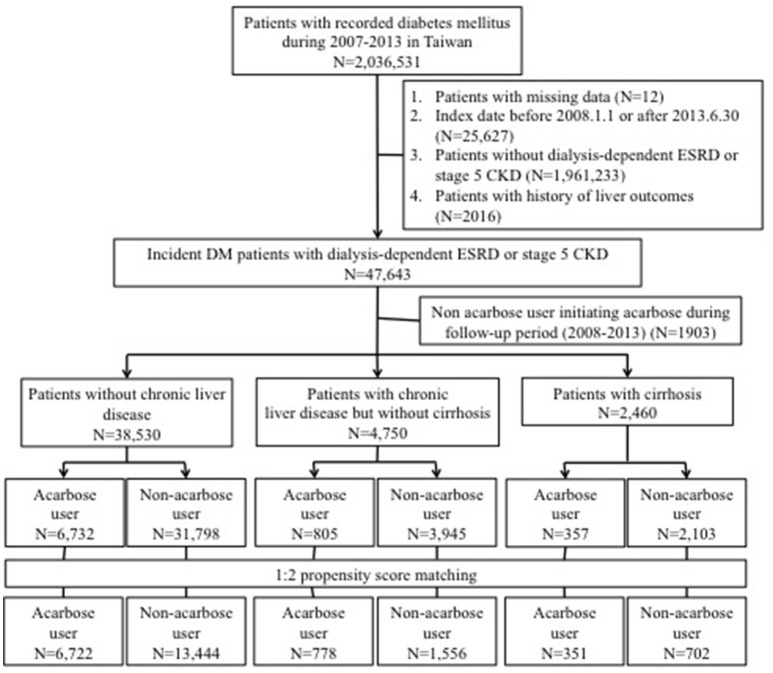
Flow chart of the current study. CKD, chronic kidney disease; DM, diabetes mellitus; ESRD, end-stage renal disease.

All adults (≥20 years old) diagnosed with DM according to the International classification of disease 9th revision—clinical modification [ICD-9-CM] codes 250.x and defined as having ≥2 discrete out-patient clinics or ≥1 hospitalization with a diagnosis of DM were systemically identified from the National Health Insurance Research Database between the study period, i.e., January 1, 2007 to December 31, 2013 (Figure 1). Because the National Health Insurance provides healthcare coverage for ≥99% Taiwanese citizens and ≥97% hospitals or clinics are in contract with this national insurance system, the database contains unequivocally the most comprehensive records of healthcare service in this country. The index date was defined as the moment when patients reached the pre-defined criteria of having DM.

Participants with DM and non-dialysis stage 5 CKD or dialysis-dependent ESRD were selected after excluding (1) those with missing data, with an index date before January 1, 2008 (to reassure incident DM cases) or after June 30, 2013 (to allow at least 6 months of follow-up); (2) those with a history of hepatotoxic injuries (the outcome of interest) before the index date; (3) or those subsequently assigned to non-users but started acarbose during follow-up. We used criteria validated by the existing literature to screen for those with non-dialysis stage 5 CKD, consisting of those having a primary diagnosis of CKD (ICD-9-CM 585.x, except 585.6) in ≥2 discrete out-patient clinics or ≥1 hospitalization, and receiving erythropoiesis-stimulating agent (ESA) treatment concurrently with the CKD diagnosis (Hsau et al., 2014; Hung et al., 2015). In Taiwan, ESA can only be initiated in CKD patients with a serum creatinine higher than 6 mg/dL according to the National Health Insurance policy, and the use of this feature to identify non-dialysis stage 5 CKD patients in Taiwan have been found to be very accurate by others (Hsau et al., 2014; Shih et al., 2014; Hung et al., 2015). The identification of those with end-stage renal disease under chronic dialysis, or stage 5D CKD patients, also followed the approach detailed in the existing literature, based on the presence of ICD-9-CM code 585.6 and the co-existence of official certificates of having end-stage renal disease under chronic dialysis, issued by the National Health Insurance Bureau of Taiwan (Chao et al., 2017; Lin et al., 2017; Wu et al., 2017a). The official certificates are called “catastrophic illness certificate,” serving as a firm proof of the patients' disease status, and are regularly audited and maintained in governmental database (called “Registry of Catastrophic Illness Database”) (Lin et al., 2014, 2017; Wu et al., 2017a).

### Participant categorization and grouping

Based on existing studies, we subsequently divided diabetic participants with non-dialysis stage 5 CKD or ESRD into those with (1) cirrhosis, defined by the presence of codes 571.2, 571.5, or 571.6 in their healthcare records and the accompanying catastrophic illness certificates; (2) with chronic liver disease (CLD), including hepatitis B [070.2, 070.3, and V02.61], hepatitis C [070.7, 070.41, 070.44, 070.51, 070.54, and V02.62], or alcoholic liver disease [291, 303, 305.0, 357.5, 425.5, 571.0, 571.1, 571.2, 571.3, 980.0, and V11.3]) but without cirrhosis; and (3) those without CLD (without any of the above codes) (Figure 1; Lin et al., 2014; Setiawan et al., 2016; Fukui et al., 2017). The spectrum of CLD traditionally includes chronic hepatitis (B, C, or non-B, non-C), alcohol-related liver disease, non-alcoholic fatty liver disease, cirrhosis, and hepatocellular carcinoma (Byrd et al., 2015). Population-based surveys consistently show that chronic hepatitis B, C, and alcoholic liver disease account for most CLD cases in different countries, including Asian ones (Fung et al., 2007; Bell et al., 2008). Furthermore, among those with DM, hepatitis C and chronic hepatitis have been found to be the main CLD etiologies (Byrd et al., 2015). Consequently, we defined CLD using a combination of diagnostic codes of chronic hepatitis B, C, and alcoholic liver disease. In addition, those with cirrhosis were specifically selected for their higher liver disease severity, due to their associated complications such as portosystemic shunts which significantly increases mortality than those without cirrhosis or CLD (Simón-Talero et al., 2018). Several reports also confirm the utility of our approach, dividing participants into those without liver disease, with non-cirrhotic liver disease, and with cirrhosis, to classify liver disease severity (Saray et al., 2012; Montomoli et al., 2013).

We examined the healthcare records of all participants during the study period and extracted their medical diagnoses, comorbidities, and medication usage and calculated their Charlson comorbidity indices (Chao et al., 2012, 2017). Comorbidities were defined using the same criteria described above. Users of medications with potential hepatotoxic risk were defined as receiving more than 30 days within the preceding year before index date, with the medication list defined according to literature and Taiwan adverse drug report (ADR) reporting system (Lee, 2013; Chen et al., 2014).

Because the severity of DM can potentially influence the risk of developing hepatic injury (Adams et al., 2017), it is important to balance this feature within the current cohort. We defined diabetic severity using the adapted diabetes complications severity index (aDCSI), a widely used framework based on claims data without laboratory parameters (Chang et al., 2012). In brief, aDCSI was derived after totaling the severity (aDCSI scores; range 0–13) or the number of 7 diabetic complications (aDCSI counts; range 0–7). The results exhibited good associations with patient outcomes (Chang et al., 2012).

### Exposures and primary outcomes

The main exposure of this study is acarbose use prior to enrollment, defined as receiving more than 30 days within the preceding year before index date. A sensitivity analysis excluding users who discontinued acarbose after index date, defined as those who did not receive any acarbose prescription after enrollment, was also carried out.

The primary outcome of this study was the development of drug-induced liver injuries (DILI) during follow-up. According to international consensus, DILI should be diagnosed based on clinical chemistry criteria, after excluding other diseases and being implicated as plausible candidates (Aithal et al., 2011). However, DILI events can be very rare, and using data from any single institute may suffer from a low yield rate, while using the administrative database for identifying DILI cases may have the advantage of higher yield rate. We defined the case as the presence of any of the following diagnosis based on the literature: bilirubin excretion disorders (277.4), toxic hepatitis (573.3), other specified or unspecified hepatic disorder (573.8, 573.9), acute/subacute hepatic necrosis (570), other biliary tract disorders (576.8), and non-neonatal unspecified jaundice (782.4), using code combinations deriving from prior reports (Kao et al., 2014; Chang et al., 2015). Based on the existing reports of acarbose-related liver injury (Kao et al., 2016), assuming that the incidence of any liver injury event in the control during follow-up was 2–3%, with a significance level of 0.05 and a power of 0.8, at least 1,859 patients would be needed per group.

### Statistical analysis

We first grouped the identified diabetic participants with stage 5 CKD or dialysis-dependent ESRD into those without CLD, with CLD but without cirrhosis, and with cirrhosis. After selecting all acarbose users from each group, propensity score-matched non-acarbose users with a 1:2 ratio were further selected for subsequent analysis, with propensity scores constructed based upon logistic regression incorporating age, sex, comorbidities, diabetic severity, and concurrent medications. Matching was done using the nearest neighbor approach, which was found to increase precision in cohort studies (Rassen et al., 2012). We compared the baseline clinical features between users and non-users using independent *t*-tests. The incidence density of liver injuries was estimated by dividing event counts with follow-up lengths (Chen et al., 2014). We then used Cox proportional hazard modeling to calculate the hazard ratios (HRs) with 95% confidence intervals (CIs) of acarbose use regarding liver injuries incidence in each group with different severities of underlying hepatic diseases. Kaplan–Meier method was used to construct event-free curves in each group, with comparisons done using a log-rank test. All statistical analyses were done using SAS software (SAS institute, Cary, NC, USA), and *p*-values lower than 0.05 were considered significant.

## Results

Among all adults with a DM diagnosis during the study period (*n* = 2,036,531), 47,643 patients with incident DM and stage 5 CKD or ESRD were identified and subsequently divided into those without CLD (84.2%), with CLD but without cirrhosis (10.4%), and with cirrhosis (5.4%) (Figure 1). A total of 17.5, 16.9, and 14.5% were acarbose users in the without, with CLD, and cirrhotic groups, respectively. We selected a 1:2 propensity score-matched ratio of users and non-users from each group for subsequent analysis. No significant differences in age, sex, all comorbidities, Charlson comorbidity index, DM severity, and other potential hepatotoxic medication were noted between acarbose users and non-users among the three groups, except hypertension (Table 1). The prevalence of hepatic, biliary, and pancreatic cancer among the three groups of patients were relatively low (without, 0.1%; mild, 5.6%; severe, 17.9%) and did not differ between users and non-users.

**Table 1:** Baseline characteristics of study participants after the process of propensity-score matching.

**Variables**	**Without CLD**			**With CLD but without cirrhosis^a^**			**With cirrhosis^b^**		
	**User (*n* = 6,722)**	**Non-user (*n* = 13,444)**	***p*-value**	**User (*n* = 778)**	**Non-user (*n* = 1,556)**	***p*-value**	**User (*n* = 351)**	**Non-user (*n* = 702)**	***p*-value**
**DEMOGRAPHIC PROFILES**									
Age (years)	66.3 ± 11.8	66.3 ± 12.5	0.86	66 ± 11.4	65.8 ± 12.6	0.78	65.3 ± 11.1	65.6 ± 11.6	0.67
Female (%)	3,156 (47)	6,274 (46.7)	0.7	330 (42.4)	643 (41.3)	0.61	140 (39.9)	297 (42.3)	0.45
**COMORBIDITIES**									
Hypertension (%)	6,545 (97.4)	13,168 (97.9)	0.01	758 (97.4)	1,525 (98)	0.37	333 (94.9)	668 (95.2)	0.84
Hyperlipidemia (%)	4,233 (63)	8,496 (63.2)	0.76	454 (58.4)	905 (58.2)	0.93	165 (47)	324 (46.2)	0.79
Atrial fibrillation (%)	806 (12)	1,586 (11.8)	0.69	90 (11.6)	170 (10.9)	0.64	41 (11.7)	81 (11.5)	0.95
COPD (%)	546 (8.1)	1,124 (8.4)	0.56	80 (10.3)	148 (9.5)	0.55	36 (10.3)	70 (10)	0.88
Acute coronary syndrome (%)	2,805 (41.7)	5,541 (41.2)	0.49	324 (41.6)	628 (40.4)	0.55	119 (33.9)	250 (35.6)	0.58
Cerebrovascular disease (%)	2,019 (30)	4,069 (30.3)	0.74	215 (27.6)	426 (27.4)	0.9	97 (27.6)	198 (28.2)	0.85
**CANCER**									
Hepatic, biliary, pancreatic (%)	4 (0.1)	10 (0.1)	0.71	43 (5.5)	88 (5.7)	0.9	60 (17.1)	128 (18.2)	0.65
Others (%)	546 (8.1)	1,068 (7.9)	0.66	84 (10.8)	172 (11.1)	0.85	35 (10)	68 (9.7)	0.88
Thyroid disorders (%)	265 (3.9)	521 (3.9)	0.82	38 (4.9)	70 (4.5)	0.68	8 (2.3)	12 (1.7)	0.52
HIV infection (%)	2 (< 0.1)	5 (< 0.1)	0.79	0 (0)	1 (0.1)	0.48	0 (0)	0 (0)	
Liver cirrhosis (%)	0 (0)	0 (0)		0 (0)	0 (0)		0 (0)	0 (0)	
HBV infection (%)	0 (0)	0 (0)		174 (22.4)	344 (22.1)	0.89	82 (23.4)	153 (21.8)	0.56
HCV infection (%)	0 (0)	0 (0)		237 (30.5)	460 (29.6)	0.65	110 (31.3)	225 (32.1)	0.82
Alcoholism (%)	0 (0)	0 (0)		43 (5.5)	88 (5.7)	0.9	62 (17.7)	118 (16.8)	0.73
Charlson comorbidity index	6.3 ± 1.9	6.3 ± 2.0	0.83	6.9 ± 2.2	6.9 ± 2.4	0.71	8.3 ± 2.2	8.2 ± 2.5	0.68
**SEVERITY OF DIABETIC COMPLICATIONS**									
aDCSI scores	3.6 ± 1.6	3.6 ± 1.6	0.4	3.6 ± 1.5	3.6 ± 1.6	0.8	3.4 ± 1.6	3.5 ± 1.6	0.59
aDCSI counts	2.1 ± 1	2.0 ± 1	0.79	2.1 ± 0.9	2.1 ± 1	0.85	2.0 ± 1	2.0 ± 1	0.83
**MEDICATIONS WITH POTENTIAL HEPATIC INFLUENCES**									
Statin (%)	3,984 (59.3)	7,890 (58.7)	0.43	381 (49)	761 (48.9)	0.98	122 (34.8)	249 (35.5)	0.82
Fibrate (%)	760 (11.3)	1,429 (10.6)	0.15	97 (12.5)	180 (11.6)	0.53	22 (6.3)	38 (5.4)	0.57
Isoniazid (%)	39 (0.6)	72 (0.5)	0.69	4 (0.5)	7 (0.4)	0.83	0 (0)	0 (0)	
Rifampicin (%)	1 (< 0.1)	2 (< 0.1)	1.00	1 (0.1)	2 (0.1)	1.00	0 (0)	0 (0)	
Trimethoprim (%)	56 (0.8)	116 (0.9)	0.83	10 (1.3)	21 (1.3)	0.9	2 (0.6)	3 (0.4)	0.75
Sulfamethoxazole (%)	729 (10.8)	1,455 (10.8)	0.96	78 (10)	158 (10.2)	0.92	31 (8.8)	59 (8.4)	0.82
Itraconazole (%)	0 (0)	0 (0)		0 (0)	0 (0)		1 (0.3)	1 (0.1)	0.62
Ketoconazole (%)	19 (0.3)	39 (0.3)	0.93	2 (0.3)	2 (0.1)	0.48	1 (0.3)	2 (0.3)	1.00
Amoxicillin (%)	5 (0.1)	11 (0.1)	0.86	0 (0)	0 (0)		0 (0)	0 (0)	
Ciprofloxacin (%)	50 (0.7)	95 (0.7)	0.77	9 (1.2)	24 (1.5)	0.46	1 (0.3)	4 (0.6)	0.53
Azithromycin (%)	5 (0.1)	11 (0.1)	0.86	2 (0.3)	0 (0)	0.05	0 (0)	0 (0)	
Phenytoin (%)	55 (0.8)	118 (0.9)	0.67	11 (1.4)	22 (1.4)	1.00	4 (1.1)	10 (1.4)	0.7
Valproic acid (%)	7 (0.1)	14 (0.1)	1.00	0 (0)	0 (0)		0 (0)	0 (0)	
Carbamazepine (%)	86 (1.3)	182 (1.4)	0.66	11 (1.4)	17 (1.1)	0.5	4 (1.1)	10 (1.4)	0.7
Diclofenac (%)	362 (5.4)	700 (5.2)	0.59	48 (6.2)	99 (6.4)	0.86	23 (6.6)	48 (6.8)	0.86
Etodolac (%)	53 (0.8)	85 (0.6)	0.2	5 (0.6)	11 (0.7)	0.86	2 (0.6)	3 (0.4)	0.75
Propylthiouracil (%)	12 (0.2)	23 (0.2)	0.9	0 (0)	0 (0)		0 (0)	0 (0)	
Methyldopa (%)	52 (0.8)	96 (0.7)	0.64	8 (1)	18 (1.2)	0.78	1 (0.3)	6 (0.9)	0.28
Acetaminophen (%)	1,656 (24.6)	3,299 (24.5)	0.88	223 (28.7)	435 (28)	0.72	103 (29.3)	194 (27.6)	0.56
Amiodarone (%)	165 (2.5)	328 (2.4)	0.95	21 (2.7)	41 (2.6)	0.93	9 (2.6)	19 (2.7)	0.89

a *With pre-existing chronic hepatitis or alcoholic liver disease (including ICD-9-CM codes 070.2, 070.3, 070.41, 070.44, 070.51, 070.54, 070.7, V02.61, V02.62, 291, 303, 305, 357.5, 425.5, 571.0, 571.1, 571.3, 980.0, V11.3)*.

b *Including ICD-9-CM codes 571.2, 571.5, 571.6 with certificates of catastrophic illness*.

*aDCSI, adapted diabetes complications severity index; CLD, chronic liver disease; COPD, chronic obstructive pulmonary disease; HBV, hepatitis B virus; HCV, hepatitis C virus; HIV, human immunodeficiency virus*.

**Figure 2:**
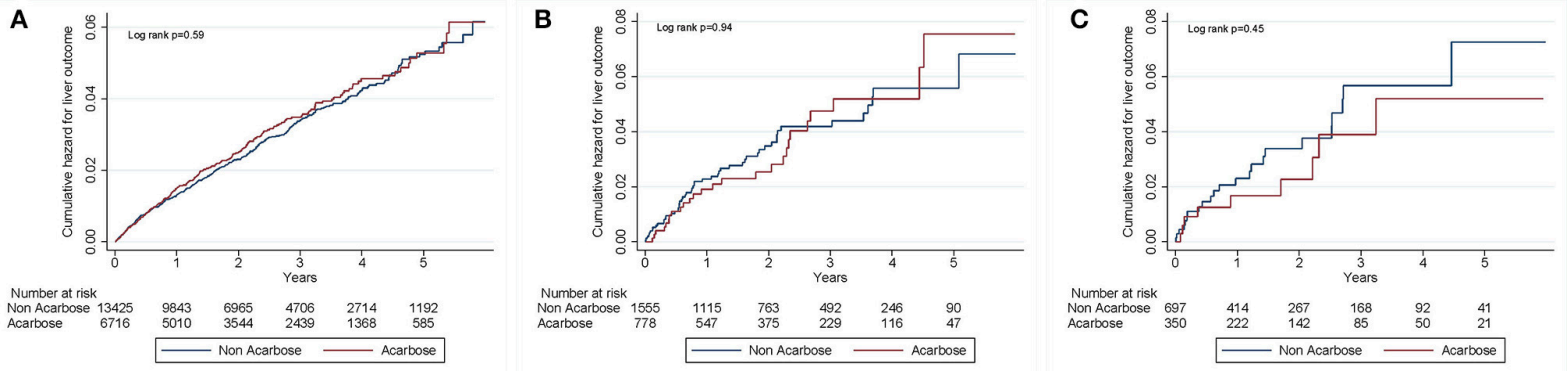
Kaplan–Meier curves for cumulative hepatotoxicity hazard during follow-up among patients without CLD **(A)**, with CLD but without cirrhosis **(B)**, and with cirrhosis **(C)**. CLD, chronic liver disease.

After a mean 2.4 years of follow-up, 661 events of liver injury occurred during 55,517.5 person-year, yielding an incidence density of 11.9 per 1,000 person-year (Table 2). Among those without CLD, the incidence of liver injury was similar among acarbose users and non-users (HR: 1.05; 95% CI: 0.88–1.25). After adjusting for demographic profiles, comorbidities, potentially hepatotoxic medication use, and aDCSI, acarbose users still exhibited a similar risk of liver injury compared to non-users (HR: 1.04–1.05) (Table 2). Among those with CLD but without cirrhosis, acarbose users also exhibited a similar risk of liver injury compared to non-users (HR: 0.97–1.00). Similar results were obtained in those with cirrhosis (Table 2). We further divided the severity of cirrhosis into those with uncomplicated cirrhosis (without esophageal varices [EV] or ascites), with compensated cirrhosis (with either EV or ascites), and those with decompensated cirrhosis (with both EV and ascites), according to past studies (Ratib et al., 2015). We found that acarbose use was not associated with a higher risk of liver injury in those with uncomplicated, compensated, and decompensated cirrhosis than non-users (for uncomplicated, HR 0.71, 95% CI 0.3–0.65; for compensated, HR 0.3, 95% CI 0.08–1.36; for decompensated, HR 1.94, HR 0.17–22) (Table 3). The cumulative hazard curves for liver injury using the Kaplan–Meier method showed that acarbose use was associated with a similar risk of liver injury compared to non-users among diabetic patients with advanced CKD/ESRD without CLD (*p* = 0.59), with CLD without cirrhosis (*p* = 0.94), and with cirrhosis (*p* = 0.45; Figure 2).

**Table 2:** Incidence and hazard ratio of hepatotoxicity events among type 2 diabetic participants with non-dialysis stage 5 CKD and Dialysis-dependent ESRD after matching.

**Variables**	**Number of events**	**Person-year**	**Incidence density^d^**	**Crude HR**		**Model A^a^**		**Model B^b^**		**Model C^c^**	
				**HR**	**95% CI**	**HR**	**95% CI**	**HR**	**95% CI**	**HR**	**95% CI**
**PATIENTS WITHOUT CLD**											
Acarbose non-users	359	32,087.7	11.2	1.00	–	1.00	–	1.00	–	1.00	–
Acarbose users	191	16,280.6	11.7	1.05	0.88–1.25	1.04	0.87–1.24	1.05	0.88–1.25	1.04	0.88–1.25
**PATIENTS WITH CLD BUT WITHOUT CIRRHOSIS**											
Acarbose non-users	53	3,444.8	15.4	1.00	–	1.00	–	1.00	–	1.00	–
Acarbose users	26	1,714.5	15.2	0.98	0.61–1.57	1.00	0.63–1.61	0.97	0.61–1.57	0.97	0.61–1.56
**PATIENTS WITH CIRRHOSIS**											
Acarbose non-users	23	1,304.3	17.6	1.00	–	1.00	–	1.00	–	1.00	–
Acarbose users	9	685.6	13.1	0.74	0.34–1.61	0.73	0.34–1.59	0.71	0.33–1.54	0.71	0.33–1.54

a *Adjusted for age, gender, comorbidity, and medications*.

b *Adjusted for age, gender, Charlson comorbidity index, and medications*.

c *Adjusted for age, gender, aDCSI, and medications*.

d *Per 1,000 person-year*.

*CI, confidence interval; CKD, chronic kidney disease; CLD, chronic liver disease; ESRD end-stage renal disease; HR, hazard ratio*.

**Table 3:** Analyses focusing only on those with cirrhosis.

**Variables**	**Number of events**	**Person-year**	**Incidence density^d^**	**Crude HR**		**Model A^a^**		**Model B^b^**		**Model C^c^**	
				**HR**	**95% CI**	**HR**	**95% CI**	**HR**	**95% CI**	**HR**	**95% CI**
**PATIENTS WITHOUT UNCOMPLICATED CIRRHOSIS**											
Acarbose non-users	50	2,674.2	18.7	1.00	–	1.00	–	1.00	–	1.00	–
Acarbose users	6	458.6	13.1	0.71	0.3–1.65	0.77	0.33–1.84	0.74	0.32–1.74	0.73	0.31–1.71
**PATIENTS WITH COMPENSATED CIRRHOSIS**											
Acarbose non-users	30	1,010.8	29.7	1.00	–	1.00	–	1.00	–	1.00	–
Acarbose users	2	215.8	9.3	0.33	0.08–1.36	0.3	0.07–1.33	0.31	0.07–1.35	0.33	0.08–1.43
**PATIENTS WITH DECOMPENSATED CIRRHOSIS**											
Acarbose non-users	1	125.1	8.0	1.00	–	1.00	–	1.00	–	1.00	–
Acarbose users	2	27.5	72.8	1.94	0.17–22	–	–	–	–	–	–

a *Adjusted for age, gender, comorbidity, and medications*.

b *Adjusted for age, gender, Charlson comorbidity index, and medications*.

c *Adjusted for age, gender, aDCSI, and medications*.

d *Per 1,000 person-year*.

*CI, confidence interval; HR, hazard ratio*.

We used defined daily dose (DDD) between 1 year prior to index date and the end of follow-up, to estimate the dose-response relationship. Categorizing the daily dose into three tertiles, we found that none of the acarbose users from each DDD tertile had an increased risk of liver injury event compared to non-users (Table 4). We also subdivided participants into those using acarbose as monotherapy and those using acarbose combined with other OADs for analyses. We found that among those using acarbose as monotherapy, users of the non-CLD, CLD without cirrhosis, and the cirrhosis groups were not at an increased risk of liver injury compared to non-users (for non-CLD, HR 0.88, 95% CI 0.56–1.38; for CLD without cirrhosis, HR 0.38, 95% CI 0.1–1.46; for cirrhosis, HR could not be calculated due to no event in the user group) (Table 5). Among those who used acarbose with other OADs, the results were unaltered (for non-CLD, HR 1.08, 95% CI 0.9–1.31; for CLD without cirrhosis, HR 1.2, 95% CI 0.72–2.01; for cirrhosis, HR 0.8, 95% CI 0.36–1.75; Table 5).

**Table 4:** Using defined daily dose (DDD) for evaluating the dose-effect relationship.

**Variables**	**Number of events**	**Person-year**	**Incidence density^d^**	**Crude HR**		**Model A^a^**		**Model B^b^**		**Model C^c^**	
				**HR**	**95% CI**	**HR**	**95% CI**	**HR**	**95% CI**	**HR**	**95% CI**
**PATIENTS WITHOUT CLD**											
Acarbose non-users	359	32,087.7	11.2	1.00	–	1.00	–	1.00	–	1.00	–
Tertile 1 (lowest)	56	5,017.6	11.2	0.99	0.75–1.31	0.99	0.75–1.32	0.99	0.75–1.31	0.99	0.74–1.31
Tertile 2	71	4,822.9	14.7	1.31	1.02–1.69^*^	1.29	1.00–1.67	1.30	1.01–1.68^*^	1.31	1.01–1.68^*^
Tertile 3 (highest)	64	6,440.1	9.9	0.9	0.69–1.17	0.89	0.68–1.16	0.90	0.69–1.17	0.89	0.68–1.1
**PATIENTS WITH CLD BUT WITHOUT CIRRHOSIS**											
Acarbose non-users	53	3,444.75	15.4	1.00	–	1.00	–	1.00	–	1.00	–
Tertile 1 (lowest)	7	528.9	13.2	0.84	0.38–1.85	0.85	0.39–1.89	0.86	0.39–1.89	0.86	0.39–1.90
Tertile 2	8	559.8	14.3	0.93	–	0.95	0.45–2.02	0.87	0.41–1.84	0.87	0.41–1.84
Tertile 3 (highest)	11	625.8	17.6	1.16	–	1.18	0.62–2.28	1.17	0.61–2.25	1.17	0.61–2.25
**PATIENTS WITH CIRRHOSIS**											
Acarbose non-users	23	1,304.3	17.6	1.00	–	1.00	–	1.00	–	1.00	–
Tertile 1 (lowest)	3	187	16.0	0.87	0.34–1.61	1.04	0.30–3.57	0.99	0.30–3.35	0.99	0.29–3.34
Tertile 2	2	189.1	10.6	0.58	–	0.63	0.14–2.75	0.54	0.13–2.29	0.54	0.13–2.29
Tertile 3 (highest)	4	309.5	12.9	0.77	–	0.64	0.22–1.88	0.68	0.23–1.98	0.68	0.23–1.98

a *Adjusted for age, gender, comorbidity, and medications*.

b *Adjusted for age, gender, Charlson comorbidity index, and medications*.

c *Adjusted for age, gender, aDCSI, and medications*.

d *Per 1,000 person-year*.

* *p < 0.05*.

*CI, confidence interval; CLD, chronic liver disease; HR, hazard ratio*.

**Table 5:** Results from dividing users into acarbose monotherapy or those using acarbose combined with other OADs.

**Variables**	**Number of events**	**Person-year**	**Incidence density^d^**	**Crude HR**		**Model A^a^**		**Model B^b^**		**Model C^c^**	
				**HR**	**95% CI**	**HR**	**95% CI**	**HR**	**95% CI**	**HR**	**95% CI**
**ACARBOSE AS MONOTHERAPY**											
**Patients without chronic liver disease**											
Acarbose non-users	64	4,113.1	15.6	1.00	–	1.00	–	1.00	–	1.00	–
Acarbose users	28	2,085.6	13.4	0.87	0.56–1.35	0.88	0.56–1.38	0.88	0.56–1.37	0.87	0.55–1.36
**Patients with chronic liver disease but without cirrhosis**											
Acarbose non-users	13	465.9	27.9	1.00	–	1.00	–	1.00	–	1.00	–
Acarbose users	3	288.8	10.4	0.38	0.11–1.34	0.38	0.10–1.46	0.40	0.11–1.46	0.44	0.12–1.63
**Patients with cirrhosis**											
Acarbose non-users	1	146.3	6.8	1.00	–	1.00	–	1.00	–	1.00	–
Acarbose users	0	113.5	0.0	–	–	–	–	–	–	–	–
**ACARBOSE COMBINED WITH OTHER** ***OADs***											
**Patients without chronic liver disease**											
Acarbose non-users	295	27,974.6	10.5	1.00	–	1.00	–	1.00	–	1.00	–
Acarbose users	163	14,195.1	11.5	1.09	0.9–1.32	1.08	0.90–1.31	1.09	0.90–1.32	1.09	0.90–1.32
**Patients with chronic liver disease but without cirrhosis**											
Acarbose non-users	40	2,978.8	13.4	1.00	–	1.00	–	1.00	–	1.00	–
Acarbose users	23	1,425.7	16.1	1.19	0.71–1.98	1.20	0.72–2.01	1.19	0.71–1.99	1.19	0.71–2.00
**Patients with cirrhosis**											
Acarbose non-users	22	1,158	19.0	1.00	–	1.00	–	1.00	–	1.00	–
Acarbose users	9	572.1	15.7	0.82	0.38–1.77	0.80	0.36–1.75	0.77	0.36–1.69	0.77	0.36–1.69

a *Adjusted for age, gender, comorbidity, and medications*.

b *Adjusted for age, gender, Charlson comorbidity index, and medications*.

c *Adjusted for age, gender, aDCSI, and medications*.

d *Per 1,000 person-year*.

*CI, confidence interval; HR, hazard ratio; OAD, oral anti-diabetic agents*.

We performed several sensitivity analyses to verify our findings. We first excluded acarbose users who discontinued acarbose after enrollment, and the results were essentially the same (Table 6). After further polishing the definition of liver injury by excluding those with alternative attributable diagnoses including autoimmune hepatitis (571.42) and shock (785.5x), we found that among patients without CLD, with CLD but without cirrhosis, or with cirrhosis, acarbose use was not associated with an increased risk of liver injury (for non-CLD, HR 1.09, 95% CI 0.93–1.28; for CLD without cirrhosis, HR 0.84, 95% CI 0.56–1.28; for cirrhosis, HR 0.61, 95% CI 0.29–1.27) (Table 7). We also narrowed down outcome definition by including only those with DILI who discontinued acarbose immediately after DILI occurred, and derived similar findings (for non-CLD, HR 0.88, 95% CI 0.73–1.06; for CLD without cirrhosis, HR 0.88, 95% CI 0.54–1.44; for cirrhosis, HR 0.58, 95% CI 0.25–1.38; Table 8). Finally, we modified our definition of acarbose exposure by using stricter definitions of acarbose exposure, focusing on those receiving more than 30 days of acarbose within 6 or 3 months preceding the index date. We found that if we used the definition of 6-month, acarbose users were not at an increased risk of liver injury than non-users among the three groups of patients (for non-CLD, HR 0.97, 95% CI 0.74–1.28; for CLD without cirrhosis, HR 1.90, 95% CI 0.88–4.10; for cirrhosis, HR 0.89, 95% CI 0.22–3.75; Table 9). Using 3-month definition, acarbose users were still not at an increased risk of liver injury than non-users among the three groups of patients (for non-CLD, HR 1.04, 95% CI 0.81–1.32; for CLD without cirrhosis, HR 1.15, 95% CI 0.58–2.30; for cirrhosis, HR 0.61, 95% CI 0.17–2.26; Table 9).

**Table 6:** Sensitivity analyses excluding acarbose users who discontinued acarbose after enrollment.

**Variables**	**Number of events**	**Person-year**	**Incidence density^d^**	**Crude HR**		**Model A^a^**		**Model B^b^**		**Model C^c^**	
				**HR**	**95% CI**	**HR**	**95% CI**	**HR**	**95% CI**	**HR**	**95% CI**
**PATIENTS WITHOUT CLD**											
Acarbose non-users	128	12,097.7	10.6	1.00	–	1.00	–	1.00	–	1.00	–
Acarbose users	83	7,033.3	11.8	1.12	0.85–1.47	1.10	0.83–1.45	1.11	0.84–1.46	1.10	0.83–1.45
**PATIENTS WITH CLD BUT WITHOUT CIRRHOSIS**											
Acarbose non-users	20	1,284.4	15.6	1.00	–	1.00	–	1.00	–	1.00	–
Acarbose users	11	704.3	15.6	1.00	0.48–2.09	1.02	0.48–2.17	1.00	0.48–2.1	1.00	0.47–2.09
**PATIENTS WITH CIRRHOSIS**											
Acarbose non-users	6	428.3	14.0	1.00	–	1.00	–	1.00	–	1.00	–
Acarbose users	4	281.8	14.2	0.99	0.28–3.53	0.32	0.06–1.85	0.65	0.16–2.59	0.65	0.16–2.59

a *Adjusted for age, gender, comorbidity, and medications*.

b *Adjusted for age, gender, Charlson comorbidity index, and medications*.

c *Adjusted for age, gender, aDCSI, and medications*.

d *Per 1,000 person-year*.

*CI, confidence interval; CLD, chronic liver disease; HR, hazard ratio*.

**Table 7:** Sensitivity analyses excluding participants with alternative attributable diagnoses (propensity-score re-matched).

**Variables**	**Number of events**	**Person-year**	**Incidence density^d^**	**Crude HR**		**Model A^a^**		**Model B^b^**		**Model C^c^**	
				**HR**	**95% CI**	**HR**	**95% CI**	**HR**	**95% CI**	**HR**	**95% CI**
**PATIENTS WITHOUT CLD**											
Acarbose non-users	852	76,654.8	11.1	1.00	–	1.00	–	1.00	–	1.00	–
Acarbose users	186	15,839.1	11.7	1.06	0.88–1.25	1.09	0.93–1.28	1.07	0.92–1.26	1.08	0.92–1.27
**PATIENTS WITH CLD BUT WITHOUT CIRRHOSIS**											
Acarbose non-users	148	8,276.4	17.9	1.00	–	1.00	–	1.00	–	1.00	–
Acarbose users	27	1,732	15.6	0.88	0.61–1.57	0.84	0.56–1.28	0.85	0.56–1.29	0.87	0.57–1.32
**PATIENTS WITH CIRRHOSIS**											
Acarbose non-users	74	3,593	20.6	1.00	–	1.00	–	1.00	–	1.00	–
Acarbose users	8	665	12.0	0.60	0.34–1.61	0.61	0.29–1.27	0.61	0.29–1.26	0.60	0.29–1.25

a *Adjusted for age, gender, comorbidity, and medications*.

b *Adjusted for age, gender, Charlson comorbidity index, and medications*.

c *Adjusted for age, gender, aDCSI, and medications*.

d *Per 1,000 person-year*.

*CI, confidence interval; CLD, chronic liver disease; HR, hazard ratio*.

**Table 8:** Sensitivity analyses in which only those who discontinued acarbose immediately after developing liver injury were selected in the acarbose group.

**Variables**	**Number of events**	**Person-year**	**Incidence density^d^**	**Crude HR**		**Model A^a^**		**Model B^b^**		**Model C^c^**	
				**HR**	**95% CI**	**HR**	**95% CI**	**HR**	**95% CI**	**HR**	**95% CI**
**PATIENTS WITHOUT CLD**											
Acarbose non-users	359	31,938.2	11.2	1.00	–	1.00	–	1.00	–	1.00	–
Acarbose users	162	16,251.7	10.0	0.89	0.74–1.07	0.88	0.73–1.06	0.88	0.73–1.06	0.88	0.73–1.06
**PATIENTS WITH CLD BUT WITHOUT CIRRHOSIS**											
Acarbose non-users	53	3,425.8	15.5	1.00	–	1.00	–	1.00	–	1.00	–
Acarbose users	23	1,709.5	13.5	0.87	0.53–1.41	0.88	0.54–1.44	0.86	0.52–1.4	0.85	0.52–1.4
**PATIENTS WITH CIRRHOSIS**											
Acarbose non-users	22	1,295.6	17.0	1.00	–	1.00	–	1.00	–	1.00	–
Acarbose users	7	681.7	10.3	0.60	0.26–1.41	0.58	0.25–1.38	0.56	0.24–1.32	0.56	0.24–1.32

a *Adjusted for age, gender, comorbidity, and medications*.

b *Adjusted for age, gender, Charlson comorbidity index, and medications*.

c *Adjusted for age, gender, aDCSI, and medications*.

d *Per 1,000 person-year*.

*CI, confidence interval; CLD, chronic liver disease; HR, hazard ratio*.

**Table 9:** Sensitivity analyses using different definitions of acarbose exposure.

**Variables**	**Number of events**	**Person-year**	**Incidence density^d^**	**Crude HR**		**Model A^a^**		**Model B^b^**		**Model C^c^**	
				**HR**	**95% CI**	**HR**	**95% CI**	**HR**	**95% CI**	**HR**	**95% CI**
**MORE THAN 30 DAYS WITHIN 6 MONTHS**											
**Patients without CLD**											
Acarbose non-users	153	13,250.6	11.5	1.00	–	1.00	–	1.00	–	1.00	–
Acarbose users	77	7,048	10.9	0.95	0.72–1.25	0.97	0.74–1.28	0.96	0.73–1.26	0.95	0.73–1.26
**Patients with CLD but without cirrhosis**											
Acarbose non-users	15	1,258.7	11.9	1.00	–	1.00	–	1.00	–	1.00	–
Acarbose users	13	631.3	20.6	1.73	0.82–3.62	1.90	0.88–4.10	1.89	0.88–4.03	1.86	0.87–3.98
*Patients with cirrhosis*										
Acarbose non-users	9	414.5	21.7	1.00	–	1.00	–	1.00	–	1.00	–
Acarbose users	4	252.3	15.9	0.72	0.22–2.33	0.89	0.22–3.75	0.71	0.21–2.43	0.68	0.20–2.37
**MORE THAN 30 DAYS WITHIN 3 MONTHS**											
**Patients without CLD**											
Acarbose non-users	193	17,027.5	11.3	1.00	–	1.00	–	1.00	–	1.00	–
Acarbose users	103	8,837.5	11.7	1.03	0.81–1.31	1.04	0.81–1.32	1.03	0.81–1.31	1.02	0.80–1.29
**Patients with CLD but without cirrhosis**											
Acarbose non-users	23	1,629.4	14.1	1.00	–	1.00	–	1.00	–	1.00	–
Acarbose users	13	825.5	15.7	1.11	0.56–2.20	1.15	0.58–2.30	1.16	0.58–2.31	1.14	0.57–2.28
**Patients with cirrhosis**											
Acarbose non-users	10	557.7	17.9	1.00	–	1.00	–	1.00	–	1.00	–
Acarbose users	4	324.9	12.3	0.68	0.21–2.16	0.61	0.17–2.26	0.67	0.20–2.21	0.66	0.20–2.21

a *Adjusted for age, gender, comorbidity, and medications*.

b *Adjusted for age, gender, Charlson comorbidity index, and medications*.

c *Adjusted for age, gender, aDCSI, and medications*.

d *Per 1,000 person-year*.

*CI, confidence interval; CLD, chronic liver disease; HR, hazard ratio*.

## Discussion

Based on a nation-wide cohort followed up prospectively, we found that among diabetic patients with stage 5 CKD or ESRD, the use of acarbose was not associated with an increased risk of liver injury. This finding clearly illustrates that the theoretical pharmacokinetic concerns of acarbose use in these patients do not occur in the real-world setting, and it is likely that we might be able to administer acarbose safely even in diabetic patients with both renal and hepatic dysfunction, a subgroup in need of more therapeutic options against hyperglycemia.

According to past reports, a latency period from 2 to 6 months can exist between the initiation of acarbose and liver toxicity events among affected diabetic patients (Hsiao et al., 2006). Taken this proposed latent period into account, our findings lend support to this hypothesis by showing that the cumulative incidence curves of users and non-users nearly converged during the initial 6 months of follow-up (Figure 2). Furthermore, we extended the spectrum of their case summary by demonstrating the absence of differences in liver toxicity incidence up to 4–5 years after the latent period. In addition, ethnic difference has been suggested to play a role in affecting the risk of metabolic idiosyncrasy related acarbose hepatotoxicity (Andrade et al., 1996), and CYP2E1 could be a plausible candidate (Wang et al., 1999). Multiple studies discovered that in patients of Chinese origin, single nucleotide polymorphism of CYP2E1 influenced the risk of lupus erythematosus and different cancers (Liao et al., 2011; Wang et al., 2014). Others also revealed that distinct polymorphisms of CYP2E1 might increase the risk of anti-tuberculosis drug-induced hepatotoxicity (Wang et al., 2016). Consequently, We believe that ethnic differences in CYP2E1 activity can influence the risk of developing acarbose-related hepatotoxicity in our studies, and further pharmacogenetic analysis are needed to elucidate the exact mechanisms of our findings.

In our study, we used Taiwan National Health Insurance Research database, an administrative healthcare database covering nearly all citizens in Taiwan, suitable for identifying rare cases or events such as DILI. Prior attempts to test for the utility of ICD-9-CM codes in identifying DILI cases based upon ICD-9-CM codes combination suggesting acute liver injury with or without codes suggesting drug poisoning/overdose (Jinjuvadia et al., 2007); although liver injury code-based approach exhibited an increased sensitivity, the results were still of concern with regard to their validity. Nonetheless, due to the limitation of the claim database we used, including the lack of biochemical data to satisfy the diagnostic criteria for DILI, and the inability to ascertain temporal causal relationship between offending drug and DILI, we could only identify DILI events based on diagnostic codes combinations which have been utilized before. Kao et al., in a study addressing anti-fungal agent hepatotoxicity, defined DILI events based on ICD-9-CM codes only (Kao et al., 2014). Others also used a very similar approach to uncover statin-related liver injury from the same database we used (Chang et al., 2015). Although our approach may suffer from low specificity, the large case number of participants is expected to partially offset this methodological insufficiency. We further increased the accuracy of our approach by excluding those with autoimmune hepatitis or shock, as shown in Table 7, with the same results. Consequently, our findings might still be useful, although prospective studies are still needed for confirmation.

In this study, we found that acarbose use in diabetic individuals with advanced CKD/ESRD and liver cirrhosis was not associated with an increased incidence of liver injury (Table 2). This is particularly important, since the options of OADs for patients with DM and hepatic impairment are limited, and the selection of treatment choice is more challenging due to their tendency of developing hypoglycemia during treatment (Scheen, 2014). However, this issue is rarely addressed in the literature (Tolman et al., 2007). An effective while safe OAD in this increasing population is urgently needed. Metformin, most sulfonylureas, meglitinide, acarbose, thiazolidinedione, and DPP4i are mostly metabolized by the liver, and pharmacokinetic changes can be observed for meglitinide and DPP4i among those with different severities of hepatic impairment (Scheen, 2014). Because the metabolites of acarbose are excreted in the urine, the combination of renal and liver dysfunction casts uncertainty on the appropriateness of acarbose usage among these patients. Our findings may provide a temporary relief for physicians caring for diabetic patients with liver dysfunction, among whom acarbose can be a useful adjunct for glycemic control even in those with CKD and cirrhosis.

The incidence of liver injury in this study was comparable to those in the literature (Chen et al., 2014). A prior study tested the relationship between acarbose use and the incidence of liver injury among diabetic patients with severe CKD using a more simplistic approach (Kao et al., 2016). They compared diabetic acarbose users vs. non-users with severe CKD only, without those carrying ESRD, enrolled fewer cases in their study, and did not adjust for other hepatotoxic medications or account for background hepatic diseases explicitly (Kao et al., 2016). Our findings further strengthen their conclusions by showing that acarbose can be used safely in diabetic CKD/ESRD patients regardless of the presence or absence of CLD. We believe that the findings of this study serves to support the renaissance of acarbose as a useful adjunct in diabetic patients with stage 5 and 5D CKD. Although a randomized controlled trial is definitely needed to validate our finding, results gained from clinical practice in real-world settings are still noteworthy and can assist us in making a reasonable choice when selecting OADs for diabetic patients with multiple morbidities.

Our study has several important limitations. First, laboratory data were unavailable in the current research database, which constitutes significant barrier for fully characterizing the nature of DILI events identified. DILI is a diagnosis of exclusion, and other alternative diagnoses should be ruled out, which can be difficult to be achieved using this database due to its retrospective nature and the possibility of missing information. Using ICD-9-CM codes to identify DILI cases has been deemed problematic by some researchers (Jinjuvadia et al., 2007). However, the main criteria we used for recognizing advanced CKD, ESRD, CLD, and cirrhosis have been cross-validated with proofs of catastrophic illness certificates, prominently lowering the uncertainty in diagnosis coding. Although the incidence of liver injury in this study was comparable to others, it is still likely that delayed hepatotoxic events occur after prolonged acarbose use. Finally, our definition of acarbose exposure may not be strict enough (more than 30 days within 1 year), but our sensitivity analyses confirmed that changing this definition to more strict ones did not alter our findings. Nonetheless, the medication adherence of the participants could not be determined accurately in this claim database study, and the cumulative acarbose dose exposed for each participant was not directly assessed. Studies incorporating longer follow-up period in these patients are needed to confirm our findings.

## Author contributions

C-TC, K-LC: Study design; C-TC, JW, J-WH, and K-LC: Data analysis; C-TC, JW, J-WH, and K-LC: Article drafting. All authors approved the final version of the manuscript.

### Conflict of interest statement

The authors declare that the research was conducted in the absence of any commercial or financial relationships that could be construed as a potential conflict of interest.

## Abbreviations

aDCSI: adapted diabetes complications severity index
CI: confidence interval
CKD: chronic kidney disease
DILI: drug-induced liver injury
DKD: diabetic kidney disease
DM: diabetes mellitus
DPP4i: dipeptidyl peptidase-4 inhibitors
eGFR: estimated glomerular filtration rate
ESRD: end-stage renal disease
EV: esophageal varices
HR: hazard ratio
ICD-9-CM: international classification of disease 9th revision—clinical modification
OAD: oral anti-diabetic agent.
